# Comparison of Acute and Chronic Surgical Complications Following Robot-Assisted, Laparoscopic, and Traditional Open Radical Prostatectomy Among Men in Taiwan

**DOI:** 10.1001/jamanetworkopen.2021.20156

**Published:** 2021-08-25

**Authors:** Szu-Yuan Wu, Chia-Lun Chang, Chang-I Chen, Chung-Chien Huang

**Affiliations:** 1 Department of Food Nutrition and Health Biotechnology, Asia University College of Medical and Health Science, Taichung, Taiwan; 2 Big Data Center, Lo-Hsu Medical Foundation, Lotung Poh-Ai Hospital, Yilan, Taiwan; 3 Division of Radiation Oncology, Lo-Hsu Medical Foundation, Lotung Poh-Ai Hospital, Yilan, Taiwan; 4 Department of Healthcare Administration, Asia University College of Medical and Health Science, Taichung, Taiwan; 5 Cancer Center, Lo-Hsu Medical Foundation, Lotung Poh-Ai Hospital, Yilan, Taiwan; 6 Graduate Institute of Business Administration, Fu Jen Catholic University, Taipei, Taiwan; 7 Centers for Regional Anesthesia and Pain Medicine, Taipei Municipal Wan Fang Hospital, Taipei Medical University, Taipei, Taiwan; 8 Wan Fang Hospital, Department of Hemato-Oncology, Taipei Medical University, Taipei, Taiwan; 9 School of Medicine, College of Medicine, Department of Internal Medicine, Taipei Medical University, Taipei, Taiwan; 10 International PhD Program in Biotech and Healthcare Management, Taipei Medical University, Taipei, Taiwan; 11 Department of Health Care Administration, Taipei Medical University College of Management, Taipei, Taiwan

## Abstract

**Importance:**

Few studies have evaluated long-term surgical complications in patients with prostate cancer (PC) who receive open radical prostatectomy (ORP), laparoscopic radical prostatectomy (LRP), or robot-assisted radical prostatectomy (RARP).

**Objective:**

To examine the perioperative and postoperative surgical complications among patients with PC who underwent ORP, LRP, or RARP.

**Design, Setting, and Participants:**

This cohort study included patients who received a diagnosis of resectable PC and underwent RP between January 1 and December 31, 2015. Participants were enrolled in the Taiwan Cancer Registry. The index date was the date of surgery, and the follow-up duration was the period from the index date to December 31, 2018. Data analysis was performed in September 2020.

**Exposures:**

ORP, LRP, or RARP.

**Main Outcomes and Measures:**

Two multivariate mixed models accounting for hospital clusters were fitted to ascertain the association of RARP with treatment outcomes (ie, hospital stay, blood transfusion, postoperative pain, erectile dysfunction, urinary incontinence, and hernia); general linear regression models were used for continuous outcomes, the amount of blood transfused, and hospital stay, and logistic regression models were used for analyzing postoperative outcomes and surgical complications.

**Results:**

Of the 1407 patients included in this study, 315 (22.4%) received ORP (mean [SD] age, 66.4 [6.8] years), 276 (19.6%) received LRP (mean [SD] age, 66.8 [6.4] years), and 816 (58.0%) received RARP (mean [SD] age, 66.1 [6.7] years). Mean (SD) follow-up in the full cohort was 36.7 (4.6) months. No statistically significant differences were observed in age, clinical tumor stage, pathological tumor stage, Gleason score, Gleason grade group, preoperative prostate-specific antigen concentration, D’Amico risk classification, and hospital level. A shorter hospital stay was observed for patients undergoing RARP vs those undergoing ORP (mean [SE] difference, −1.64 [0.22] days; *P* < .001) and LRP (mean [SE] difference, −0.57 [0.23] days; *P* = .01). Patients undergoing RARP had lower odds of receiving a blood transfusion (RARP vs ORP: adjusted odds ratio [aOR], 0.25; 95% CI, 0.17-0.36; RARP vs LRP: aOR, 0.58; 95% CI, 0.37-0.91). For postoperative pain, RARP was associated with a decrease in the odds of moderate to severe postoperative pain for as long as 12 weeks compared with both ORP and LRP (eg, RARP vs LRP at week 12: aOR, 0.40; 95% CI, 0.19-0.85; *P* = .02). The aORs for RARP vs those for ORP and LRP in the third year after RP were, for erectile dysfunction, 0.74 (95% CI, 0.45-0.92) and 0.60 (95% CI, 0.36-0.98), respectively; for urinary incontinence, 0.93 (95% CI, 0.65-0.99) and 0.60 (95% CI, 0.42-0.86), respectively; and for hernia, 0.51 (95% CI, 0.31-0.84) and 0.82 (95% CI, 0.46-0.92), respectively.

**Conclusions and Relevance:**

In this study, undergoing RARP was associated with fewer acute and chronic postoperative complications than undergoing ORP or LRP.

## Key Points

**Question:**

What are the acute and long-term procedure-specific complications associated with robot-assisted radical prostatectomy (RARP), laparoscopic radical prostatectomy (LRP), and open radical prostatectomy (ORP)?

**Findings:**

In this cohort study of 1407 men undergoing RARP, ORP, or LRP, RARP was associated with fewer acute and chronic postoperative complications than ORP or LRP.

**Meaning:**

The findings suggest that RARP may lead to fewer surgical complications than other RP approaches.

## Teaser

This cohort study examines the perioperative and postoperative surgical complications among patients with prostate cancer who underwent open, laparoscopic, or robot-assisted radical prostatectomy.

## Introduction

Prostate cancer (PC) is the second most common cancer in men worldwide according to the World Health Organization GLOBOCAN database, and according to the Taiwan Cancer Registry Database (TCRD), a similar trend is noted in Taiwan.^1,2^ Furthermore, PC is the sixth major cause of male death in Taiwan.^1^ The current lifetime PC risk for men living in the United States is approximately 1 in 8,^2^ but PC incidence is highly dependent on screening using prostate-specific antigen (PSA) concentration and the number of PSA-driven biopsies. PC is more likely to develop in older men and in African American men.^1^ Approximately 6 in 10 men diagnosed with PC are 65 years or older, and it is rare in men younger than 40 years.^1,3^ Although PC treatment depends on individual circumstances, usually no treatment is required owing to its unique characteristics.^4^ When treatment is necessary, the aim is to cure or control the disease so that it has as small as possible an effect on everyday life (ie, the fewer the treatment-related complications, the better) and life expectancy is not shortened.^4^

Radical prostatectomy (RP) is the standard surgical approach for most men choosing surgery for definitive therapy of localized PC.^5,6^ Currently, 3 common surgery options are available: robot-assisted RP (RARP), laparoscopic RP (LRP), and traditional open RP (ORP).^5,6,7,8,9,10,11^ Few studies have compared empirical perioperative and postoperative outcomes of these RP types for patients with PC.^12,13,14^ Strong evidence is lacking for concluding which of the 3 surgery types has the fewest acute and chronic surgical complications. Until now, no long-term comparative study of ORP, LRP, and RARP has been conducted for monitoring pain levels and risk of chronic surgical complications, such as erectile dysfunction, urinary incontinence (UI), hernia, benign prostatic hyperplasia (BPH) symptoms, and urethral strictures. This nationwide, population-based, all-inclusive study was conducted to understand the acute and chronic surgical complications of different RP surgical techniques.

According to a 2017 practice guideline based on a joint consensus by the American Urological Association, American Society for Radiation Oncology, and Society of Urologic Oncology, which was later endorsed by the American Society of Clinical Oncology in 2018, ORP, RP, and RARP have similar cancer control.^15^ Moreover, our previous studies^5,6^ have shown no differences in oncologic outcomes, such as surgical margin and biochemical failure-free survival, among the 3 RP types. Therefore, surgical complications are an important factor to consider in decision-making by surgeons and patients with localized PC. We conducted this study to understand what acute and long-term procedure specific-complications are associated with ORP, RP, and RARP.

## Methods

### Data Source

We conducted a population-based cohort study to determine the acute or chronic surgical complications of different RP techniques. Data were extracted from the nationwide TCRD and the Taiwan National Health Insurance Research Database (NHIRD). The NHIRD was established in 1979 and contains the data of 97% of cancer cases in Taiwan.^16^ Moreover, the data are linked with encrypted patient identifiers in the NHIRD, which includes all medical claims data on disease diagnoses, procedures, drug prescriptions, demographic characteristics, and enrollment profiles of all beneficiaries.^17^ The TCRD of the Collaboration Center of Health Information Application contains detailed patient information, such as clinical stage, pathological stage, surgical margin status, surgical procedures, treatment techniques, radiotherapy, chemotherapy regimens, and follow-up visits.^5,6,18,19,20,21,22,23,24^ Our protocols were reviewed and approved by the institutional review board of Tzu-Chi Medical Foundation. Patient consent was waived because data files are deidentified by scrambling the identification codes of both patients and medical facilities and sent to the National Health Research Institutes to form the original files of NHIRD. This study followed the Strengthening the Reporting of Observational Studies in Epidemiology (STROBE) reporting guideline.

### Study Cohort

The cohort was established based on the combined data of NHIRD and TCRD, and patients who received a diagnosis of resectable PC and underwent RP between January 1 and December 31, 2015, were included in the cohort. The index date was the date of surgery. The follow-up duration was from the index date to December 31, 2018. We selected a longer follow-up time and a relatively consistent index date of the RP because obtaining a larger sample over a longer period could affect the findings for the outcome of chronic complications, given the shorter follow-up time. Every patient with PC undergoing RP needs sufficient follow-up time for chronic surgical complications to develop; in this study, follow-up time was at least 3 years. Patient diagnoses were confirmed through a review of their pathological data, and patients who received a new diagnosis of resectable PC and underwent RP were confirmed to have no other cancer or distant metastasis. The RP type we examined was surgery to remove the entire prostate gland and the surrounding lymph nodes as treatment for localized PC.^25^ The inclusion criteria were as follows: a diagnosis of resectable PC with an indication for RP, age 20 years or older, and histologically confirmed cancer of the prostate at clinical stage T1 to T4 without distant metastasis. In our study, T1 meant that the cancer was found during examination of the prostate. After pathological confirmation of PC through biopsy, patients with PC chose RP, radiotherapy, or active surveillance depending on criteria from the National Comprehensive Cancer Network risk groups and the patients’ expected survival time.^26^ Here, pathological stage T1 was defined by the presence of pathological proof in RP and TCRD records. Those with a history of cancer before PC diagnosis, unknown clinical or pathological stage, unknown D’Amico risk classification, unknown Gleason score, unknown postoperative Gleason Grade, missing data on the preoperative PSA concentration, clinical node-positive PC, and nonadenocarcinoma histology were excluded.

### Covariates and End Points

The main independent variable in this study was the RP type: ORP, LRP, or RARP. Other covariates were age, clinical and pathological T stage, Gleason score, preoperative PSA concentration in nanograms per millimeter, D’Amico risk classification, and hospital level (academic or nonacademic). An academic medical center is a tertiary care hospital that is organizationally and administratively integrated with a medical school.^18,19,27^ End points were (1) perioperative outcomes, ie, the amount and rate of blood transfusion in milliliters and hospital stay in days; (2) acute postoperative outcomes, ie, proportion of moderate or severe postoperative pain; and (3) acute or chronic surgical complications at 3 months and 1, 2, and 3 years after RP, ie, symptoms of BPH, erectile dysfunction, UI, urethral stricture, and hernia.

### Statistical Analysis

Patient characteristics were first described according to the surgical modality by using descriptive statistics, such as mean and SD for normal continuous data, median and interquartile range for nonnormal continuous data, and number and percentage for categorical data. The *t* test, analysis of variance, and their nonparametric counterpart tests were used, as appropriate. Two types of multivariate mixed models accounting for patient clusters in hospitals were fitted to ascertain the association of RARP with the outcomes: general linear regression models for continuous outcomes, the amount of blood transfused, and hospital stay, with propensity score adjustment of age, clinical T stage, pathological T stage, Gleason score, preoperative PSA concentration, D’Amico risk classification, and hospital levels, and logistic regression models for postoperative outcomes and surgical outcomes, with propensity score adjustment for age, clinical T stage, pathological T stage, Gleason score, preoperative PSA concentration, D’Amico risk classification, and hospital level. Significance levels were set at *P* < .05, all tests were 2-tailed. While fitting the mixed-effects model, we tested whether variance components (G matrix) were zero. The *P* value of the likelihood ratio test based on mixture of χ^2^ tests was less than .05, indicating that the model provided a significantly better fit compared with a model without a random intercept for hospital. All analyses were conducted using SAS version 9.3 (SAS Institute).

## Results

### Clinicopathological Characteristics

Of the 1407 patients included in this study, 315 (22.4%) received ORP (mean [SD] age, 66.4 [6.8] years), 276 (19.6%) received LRP (mean [SD] age, 66.8 [6.4] years), and 816 (58.0%) received RARP (mean [SD] age, 66.1 [6.7] years). The mean (SD) follow-up duration after the index date was 36.7 (4.6) months. No statistically significant differences were observed in age, clinical T stage, pathological T stage, Gleason score, Gleason grade group, preoperative PSA concentration, D’Amico risk classification, or hospital level (Table 1). The covariates were not statistically significant among the 3 RP types. Table 2, Table 3, and Table 4 provide the association of the RP type with perioperative and acute and chronic postoperative surgical complications.

**Table 1.** Patient Characteristics Stratified by Surgical Modality

Characteristic	Patients, No. (%)			*P* value
	ORP (n = 315)	LRP (n = 276)	RARP (n = 816)	
Age, y				
Mean (SD)	66.4 (6.8)	66.8 (6.4)	66.1 (6.7)	.47
Median (IQR)	67 (62-71)	67 (62-72)	66 (62-71)	
20-59	49 (15.6)	41 (14.9)	130 (15.9)	.90
60-69	165 (52.4)	146 (52.9)	444 (54.4)	
≥70	101 (32.1)	89 (32.2)	242 (29.7)	
cT stage				
cT1	84 (26.7)	75 (27.2)	195 (23.9)	.28
cT2	149 (47.3)	133 (48.2)	436 (53.4)	
cT3-cT4	82 (26.0)	68 (24.6)	185 (22.7)	
pT stage				
pT1	96 (30.5)	83 (30.1)	237 (29.0)	.19
pT2	152 (48.3)	137 (49.6)	432 (52.9)	
pT3a	37 (11.7)	30 (10.9)	76 (9.3)	
pT3b	30 (9.5)	26 (9.4)	71 (8.7)	
Gleason score				
2-6	34 (10.8)	37 (13.4)	142 (17.4)	.10
3 + 4	110 (34.9)	89 (32.2)	274 (33.6)	
4 + 3	62 (19.7)	53 (19.2)	160 (19.6)	
8-10	109 (34.6)	97 (35.1)	240 (29.4)	
Preoperative PSA, ng/mL				
0-5	37 (11.7)	32 (11.6)	94 (11.5)	.65
6-10	110 (34.9)	95 (34.4)	285 (34.9)	
11-20	86 (27.3)	82 (29.7)	233 (28.6)	
>20	82 (26.0)	67 (24.3)	204 (25.0)	
D’Amico risk classification				
Low	13 (4.1)	15 (5.4)	58 (7.1)	.11
Intermediate	93 (29.5)	69 (25.0)	219 (26.8)	
High	122 (38.7)	120 (43.5)	338 (41.4)	
Advanced	87 (27.6)	72 (26.1)	201 (24.6)	
Hospital level				
Academic center	258 (81.9)	225 (81.5)	673 (82.5)	.73
Nonacademic center	57 (18.1)	51 (18.5)	143 (17.5)	
Follow-up duration, mean (SD), mo	36.1 (4.4)	37.2 (5.0)	36.2 (4.7)	NA
Death	8 (2.5)	4 (1.4)	11 (1.3)	.35

Abbreviations: cT, clinical tumor; IQR, interquartile range; LRP, laparoscopic radical prostatectomy; NA, not applicable; ORP, open radical prostatectomy; PSA, prostate-specific antigen; pT, pathological tumor; RARP, robot-assisted radical prostatectomy.

SI conversion factor: To convert PSA to micrograms per liter, multiply by 1.0.

**Table 2.** General Linear Regression Analysis of BT Volume and Hospital Length of Stay Length, Stratified by Surgical Modality

Perioperative outcome	Mean (SD)			RARP vs ORP		RARP vs LRP	
	ORP (n = 315)	LRP (n = 276)	RARP (n = 816)	Mean difference (SE)^a^	*P* value	Mean difference (SE)^a^	*P* value
BT volume, mL	914.5 (927.0)	750.0 (893.2)	766.9 (978.1)	−167.16 (181.81)	.36	−46.43 (225.86)	.84
Hospital stay, d	9.4 (3.5)	8.4 (3.2)	7.8 (3.2)	−1.64 (0.22)	<.001	−0.57 (0.23)	.01

Abbreviations: BT, blood transfusion; ORP, open radical prostatectomy; LRP, laparoscopic radical prostatectomy; RARP, robot-assisted radical prostatectomy.

^a^ General linear regression for continuous variables with propensity score adjustment of age, clinical tumor stage, pathological tumor stage, Gleason score, preoperative prostate-specific antigen concentration, D’Amico risk classification, and hospital levels.

**Table 3.** Logistic Regression for the Association of Surgical Modality Types With BT Rate and Postoperative Pain

Outcome	No. (%)			RARP vs ORP		RARP vs LRP	
	ORP (n = 315)	LRP (n = 276)	RARP (n = 816)	aOR (95% CI)^a^	*P* value	aOR (95% CI)^a^	*P* value
**Perioperative outcome**							
BT incidence	76 (24.1)	35 (12.7)	59 (7.2)	0.25 (0.17-0.36)	<.001	0.58 (0.37-0.91)	.02
**Moderate postoperative pain**							
Day 1	114 (36.1)	67 (24.2)	250 (30.6)	0.84 (0.67-0.95)	.01	1.07 (0.58-1.13)	.18
Week 1	82 (26.0)	64 (23.2)	163 (20.0)	0.65 (0.35-0.82)	.03	0.62 (0.46-0.82)	<.001
Week 12	10 (3.2)	14 (5.1)	17 (2.1)	0.74 (0.33-1.67)	.46	0.40 (0.19-0.85)	.02
**Severe postoperative pain**							
Day 1	201 (63.8)	189 (68.5)	456 (55.9)	0.72 (0.55-0.86)	.01	0.69 (0.50-0.96)	.03
Week 1	138 (43.8)	139 (50.4)	342 (41.9)	0.90 (0.69-0.97)	.04	0.73 (0.55-0.97)	.03
Week 12	0	0	0	NA	NA	NA	NA

Abbreviations: aOR, adjusted odds ratio; BT, blood transfusion; LRP, laparoscopic radical prostatectomy; ORP, open radical prostatectomy; RARP, robot-assisted radical prostatectomy.

^a^ Logistic regression for binary variables with propensity score adjustment of age, clinical tumor stage, pathological tumor stage, Gleason score, preoperative prostate-specific antigen concentration, D’Amico risk classification, and hospital level.

**Table 4.** Logistic Regression for the Association of Surgical Modality Types on Acute and Chronic Surgical Complications

Outcome	Patients, No. (%)			RARP vs ORP		RARP vs LRP	
	ORP (n = 315)	LRP (n = 276)	RARP (n = 816)	aOR (95% CI)^a^	*P* value	aOR (95% CI)^a^	*P* value
**3 mo After RP**							
BPH symptoms	141 (44.8)	136 (49.3)	387 (47.4)	1.05 (0.80-1.38)	.71	0.86 (0.65-1.13)	.27
Erectile dysfunction	14 (4.4)	16 (5.8)	45 (5.5)	1.14 (0.61-2.13)	.69	0.89 (0.49-1.62)	.70
UI	53 (16.8)	66 (23.9)	150 (18.4)	1.02 (0.92-1.89)	.14	0.80 (0.57-0.94)	.03
Urethral strictures	6 (1.9)	3 (1.1)	8 (1.0)	0.55 (0.18-1.68)	.29	1.00 (0.25-3.99)	>.99
Hernias	10 (3.2)	6 (2.2)	17 (2.1)	0.56 (0.25-1.27)	.17	0.89 (0.34-2.32)	.81
**1 y After RP**							
BPH symptoms	160 (50.8)	150 (54.3)	444 (54.4)	1.10 (0.84-1.43)	.51	0.93 (0.70-1.23)	.63
Erectile dysfunction	28 (8.9)	30 (10.9)	70 (8.6)	0.88 (0.55-1.42)	.61	0.71 (0.44-1.12)	.14
UI	77 (24.4)	76 (27.5)	187 (22.9)	1.07 (0.77-1.47)	.69	0.89 (0.64-1.23)	.48
Urethral strictures	10 (3.2)	5 (1.8)	11 (1.3)	0.43 (0.17-1.08)	.07	0.80 (0.27-2.43)	.70
Hernias	31 (9.8)	23 (8.3)	65 (8.0)	0.77 (0.48-1.21)	.25	0.90 (0.54-1.49)	.67
**2 y After RP**							
BPH symptoms	146 (46.3)	118 (42.8)	350 (42.9)	0.82 (0.63-1.07)	.15	0.96 (0.73-1.28)	.80
Erectile dysfunction	29 (9.2)	40 (14.5)	70 (8.6)	0.87 (0.61-0.93)	.049	0.58 (0.38-0.88)	.01
UI	70 (22.2)	74 (26.8)	171 (21.0)	0.96 (0.76-1.47)	.74	0.82 (0.59-1.13)	.23
Urethral strictures	6 (1.9)	8 (2.9)	11 (1.3)	0.77 (0.27-2.22)	.63	0.50 (0.19-1.31)	.16
Hernias	34 (10.8)	35 (12.7)	81 (9.9)	0.95 (0.62-1.46)	.81	0.78 (0.51-1.20)	.26
**3 y After RP**							
BPH symptoms	107 (34.0)	93 (33.7)	246 (30.1)	0.79 (0.59-1.05)	.11	0.82 (0.61-1.11)	.20
Erectile dysfunction	26 (8.3)	27 (9.8)	51 (6.3)	0.74 (0.45-0.92)	.04	0.60 (0.36-0.98)	.04
UI	54 (17.1)	66 (23.9)	119 (14.6)	0.93 (0.65-0.99)	.04	0.60 (0.42-0.86)	.005
Urethral strictures	3 (1.0)	4 (1.5)	9 (1.1)	4.27 (0.48-37.70)	.19	0.47 (0.16-1.41)	.18
Hernias	30 (9.5)	17 (6.2)	44 (5.4)	0.51 (0.31-0.84)	.009	0.82 (0.46-0.92)	.02

Abbreviations: aOR, adjusted odds ratio; BPH, benign prostatic hyperplasia; LRP, laparoscopic radical prostatectomy; ORP, open radical prostatectomy; RARP, robot-assisted radical prostatectomy; RP, radical prostatectomy; UI, urinary incontinence.

^a^ Logistic regression for binary variables with propensity score adjustment of age, clinical tumor stage, pathological tumor stage, Gleason score, preoperative prostate-specific antigen concentration, D’Amico risk classification, and hospital level.

### Blood Transfusion Volume and Hospital Stay Duration, Stratified by Surgical Modality

No statistically significant differences were observed in the amount of blood transfusion received (Table 2). Patients undergoing ORP, LRP, and RARP had a mean (SD) blood transfusion volume of 914.5 (927.0) mL, 750.0 (893.3) mL, and 766.9 (978.1) mL, respectively. Those undergoing RARP had shorter hospital stay than those undergoing ORP and LRP, showing least-square mean differences in the mixed model (RARP vs ORP: mean difference [SE], −1.64 [0.22]; *P* < .001; RARP vs LRP: mean difference, −0.57 [0.23]; *P* = .01) (Table 2).

### Blood Transfusion Rate and Postoperative Pain

Overall, 76 patients undergoing ORP (24.1%), 35 patients undergoing LRP (12.7%), and 59 patients undergoing RARP (7.2%) required blood transfusions. Patients who underwent RARP had lower odds of receiving blood transfusion than those undergoing ORP or LRP (RARP vs ORP: adjusted odds ratio [aOR], 0.25; 95% CI, 0.17-0.36; RARP vs LRP: aOR, 0.58; 95% CI, 0.37-0.91) (Table 3). The number of patients experiencing moderate postoperative pain on the first day of surgery was lower in the RARP group than in the ORP group (250 [30.6%] vs 114 [36.1%]). Furthermore, there were fewer patients with moderate or severe postoperative pain in the first week after surgery in the RARP group than in both ORP and LRP groups and fewer patients with moderate pain in week 12 in the RARP group than the LRP group. Overall, patients in the RARP group had lower odds of experiencing moderate pain on postoperative day 1 (RARP vs ORP: aOR, 0.84; 95% CI, 0.67-0.95), week 1 (RARP vs ORP: aOR, 0.65; 95% CI, 0.35-0.82; RARP vs LRP: aOR, 0.62; 95% CI, 0.46-0.82), and week 12 (RARP vs LRP: aOR, 0.40; 95% CI, 0.19-0.85) as well as severe postoperative pain on day 1 (RARP vs ORP: aOR, 0.72; 95% CI, 0.55-0.86; RARP vs LRP: aOR, 0.69; 95% CI, 0.50-0.96) and week 1 (RARP vs ORP: aOR, 0.90; 95% CI, 0.69-0.97; RARP vs LRP: aOR, 0.73; 95% CI, 0.55-0.97). At week 12, no severe postoperative pain was noted among the ORP, LRP, or RARP groups.

### Acute and Chronic Surgical Complications

For all modalities, the incidence of surgical complications decreased over time. RARP had the lowest incidence of hernia, erectile dysfunction, and UI 3 years after surgery than the other modalities (hernia: RARP, 44 [5.4%]; LRP, 17 [6.2%]; ORP, 30 [9.5%]; erectile dysfunction: RARP, 51 [6.3%]; LRP, 27 [9.8%]; ORP, 26 [8.3%]; UI: RARP, 119 [14.6%]; LRP, 66 [23.9%]; ORP, 26 [8.3%]). For all the 3 modalities, the incidence of erectile dysfunction decreased between years 2 and 3 after RP. RARP was associated with a decrease in the odds of UI at 3 months after RP compared with LRP (aOR, 0.80; 95% CI, 0.57-0.94) (Table 4). In the second year after RP, RARP reduced the odds of erectile dysfunction by more than 40% compared with LRP (aOR, 0.58; 95% CI, 0.38-0.88) and significantly reduced erectile dysfunction risk compared with ORP (aOR, 0.87; 95% CI, 0.61-0.93). In year 3, the odds of chronic surgical complications, except BPH symptoms and urethral strictures, were lower in patients who received RARP compared with those who underwent either ORP or LRP. In the third year after RP, the aORs for RARP compared with those for ORP and LRP for erectile dysfunction were 0.74 (95% CI, 0.45-0.92) and 0.60 (95% CI, 0.36-0.98); for UI, 0.93 (95% CI, 0.65-0.99) and 0.60 (95% CI, 0.42-0.86); and for hernia, 0.51 (95% CI, 0.31-0.84) and 0.82 (95% CI, 0.46-0.92).

## Discussion

RP can be performed using either an open approach or a minimally invasive (robotic or laparoscopic) technique. Analyses of large databases have indicated that the performance of RARP has increased rapidly, and it now constitutes the technique used in most RPs.^12,28^ A Cochrane review of 2 randomized trials showed that minimally invasive and open prostatectomy resulted in similar urinary and sexual quality-of-life outcomes. Furthermore, overall and serious complication rates were comparable: patients had shorter hospital stay and fewer blood transfusions after minimally invasive prostatectomy than after traditional RP. However, postoperative pain and chronic postoperative complications were not examined using long-term follow-up and high-quality data.^13^ In addition to a small incision, the advantages of minimally invasive techniques mainly include favorable perioperative outcomes, such as reduced blood loss. Individual comparative studies of effect and adverse effects have not established the superiority of one approach over another, and the observed differences may reflect differences in case selection and the lack of long-term follow-up. In previous studies,^12,13,28^ minimally invasive techniques were not separately evaluated as RARP and LRP. There were retrospective comparative studies of ORP and RARP, but LRP was not included in these.^29,30^ By contrast, a retrospective study by Coughlin et al^31^ in 2018 included 326 patients with PC (whose data were extracted from another randomized clinical trial), and the follow-up duration was shorter (ie, 24 months) than in this study. In addition, the comparison performed by Coughlin et al^31^ was restricted to biochemical recurrence-free survival rates and health-related and domain-specific quality-of-life outcomes between patients with PC receiving ORP and RARP; LRP was not evaluated. Moreover, in the study by Coughlin et al,^31^ numerous confounding factors were not considered with regard to adjustment (including propensity score adjustment) for the multivariate analysis. Therefore, to our knowledge, our study is the first comparative study to balance case selection and decrease the bias of surgical approach and difficulties in RP among patients undergoing ORP, LRP, and RARP (Table 1). In addition to the perioperative complications of blood transfusion and hospital stay, we conducted long-term follow-up for chronic (ie, 3 years after RP) surgical complications (Table 2, Table 3, and Table 4). To our knowledge, this is the first study to compare the perioperative and long-term postoperative complications among patients undergoing ORP, LRP, and RARP.

In this study, the volume of blood transfusion was highest in patients undergoing ORP, followed by RARP and LRP (with 914.5 mL, 766.9 mL, and 750.0 mL, respectively), which was expected and is compatible with the findings of previous studies (Table 2).^12,13,28^ However, no significant difference was observed in the blood transfusion volume among the groups (Table 2). Conversely, significant differences were noted in the incidence of blood transfusion among the RARP, ORP, and LRP groups (Table 3). These outcomes of risk or volume of blood transfusion are compatible with the findings of previous studies.^12,13,32^ The lack of differences in the blood transfusion volume among the 3 RP techniques may be due to the hospital volume effect.^6^ In Taiwan, traditional ORP has been performed by highly experienced surgeons for many years,^5,6,33^ which may have minimized the blood volume required for transfusion. Because LRP and RARP are designed to require a low volume of blood for transfusion and because this surgery is conducted in low-volume hospitals, their transfusion volume may not be significantly different in this study.^6^ In addition, surgical outcomes have been found to be associated with hospital volume in RARP but not in nonrobotic RP techniques^6^; thus, the ORP-induced blood transfusion volume was not significantly different from that induced by LRP and RARP (Table 2).^6^ However, the mean length of hospital stay for RARP was significantly shorter than that for ORP or LRP after general linear regression for continuous variables with propensity score adjustment of age, clinical T stage, pathological T stage, Gleason score, preoperative PSA concentration, D’Amico risk classification, and hospital level (Table 2). This finding is compatible with the findings of previous studies.^12,13,14,34^ To our knowledge, our study is the leading comparative study to show that RARP has the shortest hospital stay and the lowest blood transfusion risk vs LRP and ORP, particularly in Asian patients (Table 2 and Table 3).

The percentage of patients requiring blood transfusion in this study was 24.1%, 12.7%, and 7.2% in ORP, LRP, and RARP, respectively, showing a substantial difference. To our knowledge, this is the first study to show the blood volume required and blood transfusion incidence for ORP, LRP, and RARP in patients with PC. Few reports have provided information on the volume of blood transfusion to aid physicians and patients in shared decision-making regarding the RP technique. With traditional RP, a greater risk of blood transfusion exists compared with the minimally invasive techniques of RP and ORP.^12,13,14^ Our findings suggest that RARP has lowest hemorrhage risk in perioperative complications because RARP was associated with the lowest odds of blood transfusion (Table 3). To our knowledge, this study is the first in Asia to show that RARP has the lowest risk of blood transfusion. Our study is valuable because surgical outcomes are different between Western and Eastern countries owing to different pelvic anatomies, which has resulted in different challenges in RP between Asian and White patients with PC.^5,6,33,35,36,37^

Another notable finding of this study was the differences in pain among the 3 RP techniques. To our knowledge, no study has reported on postoperative pain among the 3 RP types. Medication prescribed, ie, morphine or opioids for severe pain and nonsteroidal anti-inflammatory drugs (NSAIDs) at an average dosage of 800 mg or greater for mild to moderate pain, are surrogates for pain level. We found that pain levels of patients undergoing all 3 modalities diminished with time (Table 3). Furthermore, for moderate and severe postoperative pain, the percentages of patients taking medication in the RARP and LRP groups were lower than those in the ORP group. RARP had fewer patients with moderate postoperative pain compared with LRP for all comparisons except for the first day after surgery; fewer patients who underwent RARP had severe postoperative pain at the first day and first week than those who underwent LRP. This suggests that RARP has the smallest association with moderate or severe pain among patients in the short and long term.

According to our literature review, no comparative report is available for acute and chronic postoperative complications, such as BPH symptoms, UI, erectile dysfunction, urethral stricture, and hernia among ORP, LRP, and RARP in patients with PC. To our knowledge, this study is the first to provide valuable information on the incidence of acute and chronic postoperative complications and to perform multivariate analysis of logistic regression for variables with propensity score adjustment (Table 4). We examined acute and chronic postoperative complications at 3 months and at 1, 2, and 3 years. No significant difference was observed in BPH at 3 months, a period that usually indicates short-term complications after surgery. Moreover, no difference was found in erectile dysfunction, urethral stricture, and hernia at 3 months, as more time is required to develop these postoperative complications. However, the incidence of surgical complications gradually decreased with time for all 3 RP types (Table 4). For chronic postoperative complications, including those at 1, 2, or 3 years after RP, Table 4 presents the long-term follow-up of chronic surgical complication risks stratified by modality through logistic regression. In this study, RARP had the lowest incidence of hernia, erectile dysfunction, and UI in the third year after RP. Those who experienced erectile dysfunction in the second year showed much improvement. RARP had the lowest odds of experiencing erectile dysfunction, UI, or hernia compared with ORP and LRP. To our knowledge, no study has demonstrated long-term postoperative complications in terms of their incidence or comparative data for ORP, LRP, and RARP. In terms of surgical complications, RARP might be beneficial for perioperative outcomes (ie, pain levels, blood transfusion risk, and hospital stay) and chronic postoperative outcomes (ie, erectile dysfunction 2 years after RP; erectile dysfunction, UI, and hernia 3 years after RP) rather than subchronic postoperative outcomes (ie, 3 months or 1 year after RP). To our knowledge, this is the leading study to use real-world data to provide valuable information regarding the surgical complications to inform decision-making by physicians and patients with PC.

Furthermore, this is the leading population-based study of the association of RARP with perioperative outcomes as well as with acute or chronic postoperative complications. Although RARP was not associated with the blood volume required for transfusion, it was associated with lower odds of receiving blood transfusion, subsequent hospitalization, moderate to severe postoperative pain for as long as 12 weeks, and chronic surgical complications compared with both ORP and LRP (Table 2, Table 3, and Table 4). The strengths of this study are its sufficiently large sample size, long-term follow-up of chronic postoperative complications, and the consistent covariates of patients with PC receiving different RP techniques (Table 1). In our current study, we balanced clinicopathological characteristics among the different RP techniques to ensure consistency in the complexity and difficulties of the different RP types. Most major covariates that affect RP, such as age, clinical and pathological T stage, Gleason score, preoperative PSA, D’Amico risk classification, and hospital level, were not statistically significant (Table 1). To our knowledge, this is the leading, largest, and only long-term follow-up cohort study to estimate the perioperative and acute and chronic postoperative complications of blood transfusion volume, blood transfusion risk, hospital stay, postoperative pain, erectile dysfunction, UI, hernia, BPH symptoms, and urethral stricture among the different RP techniques. In our findings, RARP was associated with fewer perioperative and acute and chronic postoperative complications for men with localized PC than ORP and LRP. These findings should be considered in future clinical practice and prospective clinical trials.

### Limitations

This study has limitations. First, in Table 1, the controlled variables of the 3 modalities were examined, which were statistically comparable (ie, none of the *P* values were significant). Although we tried to balance known confounders to reduce bias that could affect the end points, unexpected confounders might exist for perioperative or postoperative complications. Second, because all patients in this study were Asian, the corresponding racial/ethnic susceptibility remains unclear; hence, our results should be cautiously extrapolated to non-Asian populations. Third, the use or nonuse of nerve-sparing techniques is not recorded in our database as a covariate, and this is a limitation of the analysis of postoperative sexual function. The same is true of baseline (ie, preoperative) sexual function. It is difficult to know whether the difference in sexual function is because of the robotic technique itself or greater implementation of nerve sparing. Fourth, the diagnoses of all comorbidities were based on *International Classification of Diseases, Ninth Revision, Clinical Modification* codes. Although these administrative codes are commonly used for comorbidities, they are highly problematic for determining the presence or absence of disease. However, several validation studies have been performed to evaluate the validity of diagnosis codes in the NHIRD.^38,39,40,41^ Most of the validated diagnosis codes are for common conditions or severe diseases and have modest to high sensitivity and positive predictive values (eg, epilepsy, ischemic stroke, hypertension, diabetes, hyperlipidemia, fibrillation, all cancers).^38,39,40,41^ Moreover, the Taiwan Cancer Registry Administration randomly reviews medical records and interviews patients to verify the accuracy of the diagnoses, and hospitals with outlier charges or practices may be audited and subsequently penalized if malpractice or discrepancies are identified. Therefore, to obtain crucial information on population specificity and disease occurrence, a large-scale randomized clinical trial comparing carefully selected patients undergoing suitable treatments is essential. We believe a randomized clinical trial can be done, given the generally accepted body of evidence supporting the superiority of robotic vs open prostatectomy in the modern era. Additionally, the TCRD does not contain information on dietary habits and body mass index, all of which may be risk factors for perioperative or postoperative complications. However, considering the magnitude and statistical significance of the observed association in this study, these limitations are unlikely to affect the conclusions.

## Conclusions

In this population-based cohort study of the association of RARP with perioperative or postoperative complications as well as short-term to long-term surgical complications, RARP was associated with a decrease in the odds of blood transfusion, subsequent hospitalization, moderate to severe postoperative pain for as long as 12 weeks, and chronic surgical complications compared with both ORP and LRP. In the future, RARP might be preferred by physicians and patients with PC.
